# Knowledge, beliefs and practices regarding sickle cell eye disease of patients at the sickle cell unit, Jamaica

**DOI:** 10.11604/pamj.2019.32.84.14742

**Published:** 2019-02-19

**Authors:** Lizette Mowatt, Ayodeji Ajanaku, Jennifer Knight-Madden

**Affiliations:** 1 Ophthalmology Division, Department of Surgery, Radiology, Anaesthesia and Intensive Care, Faculty of Medical Sciences, University of the West Indies, Mona, Jamaica; 2 Ophthalmology Division, University Hospital of the West Indies, Jamaica; 3 Russell Hall Hospital, The Dudley Group NHS Foundation Trust; 4 Sickle Cell Unit, Caribbean Institute for Health Research, University of the West Indies, Mona Campus Kingston 7, Jamaica

**Keywords:** Knowledge, beliefs, practices, sickle cell retinopathy, Jamaica, Sickle Cell Unit

## Abstract

**Introduction:**

Sickle cell disease can result in visually threatening eye disease (proliferative sickle cell retinopathy). This can be prevented with timely eye screening. It is important for patients to understand their role. Our research is to determine the knowledge, beliefs and practices (KBP) regarding eye disease of Sickle Cell patients and the impact of genotype, demographic and socio-economic status.

**Methods:**

Cross-sectional study at the Sickle Cell Unit, Jamaica during May 2016. Consecutive non-pregnant adults (>18 years of age) attendees, who were not acutely unwell, were invited to participate. A 26-item single interviewer administered questionnaire was used to obtain socio-demographic data, highest level of education completed, employment status, sickle cell genotype, if known, frequency of clinic attendance and patients' knowledge, beliefs and practices. Ten of these were yes/no questions, whereas eight required that they choose correct answers from four choices.

**Results:**

One hundred subjects were recruited, 72% had homozygous SS disease. Their ages ranged from 18-63 years (mean 34.1 years, SD11.3). Fifty six percent were female. Most (75%) had achieved at least secondary education. The majority (62%) were unemployed. The mean belief score was 3.6/6(60%) and the mean knowledge and practice scores were 3.3/7(47%) and 2.2/5(44%) respectively. Milder genotypes had higher knowledge scores vs the more severe genotypes (4.0 vs 3.2, P=0.013). Only 28% had regular eye examinations; less than 50% had seen an ophthalmologist in the past year. Practice scores were higher in employed than in unemployed patients (2.6 vs 1.9, (P=0.04)). Employed patients were more likely than the unemployed to see their eye doctor for regular eye “examinations” (42.1% vs 19.4%, χ2=6.0, P=0.02). The practice and knowledge scores correlated (r^2^=0.363, P<0.001) and belief score (r^2^=0.304, P =0.002), except where 98% believed they should see an ophthalmologist annually, but only 42% did, and 21% had never.

**Conclusion:**

Knowledge scores were fair, however, the practice was not always in keeping with knowledge.

## Introduction

Sickle cell disease (SCD), a hereditary blood disorder is one of the most prevalent genetic diseases worldwide, with over 300,000 new births annually [1, 2]. The highest incidence is amongst individuals with African descent, hence its prevalence in the Caribbean. However, the genotype is also prevalent in the Mediterranean, Middle East, India, the Caribbean, South and Central America [1, 2]. The sickle cell gene is present in 10.1% of Jamaicans [3]. Sickle cell retinopathy can be a blinding disease, which can be prevented with screening and treatment. In patients aged 24-26 years old, the prevalence of Proliferative Sickle Retinopathy (PSR) was 43% in persons with Haemoglobin (Hb)SC disease and 14% in persons with homozygous sickle cell (Hb SS) disease in the Jamaica Sickle Cell Cohort Study [4]. PSR can be detected by routine screening when the patient is asymptomatic and early intervention can improve outcome [5]. Awareness of risk of blindness from eye diseases (such as retinopathy) in patients who are asymptomatic, was associated with more frequent use of eye care services [6]. In Jamaicans referred to an ophthalmology clinic with diabetic retinopathy, approximately half were unaware of the recommendation to have annual eye examinations prior to their referral to the specialist clinic [7]. The impact of counselling on knowledge and beliefs may vary with demographic indices such as age, gender and socioeconomic background [6, 7]. Practice may also be affected by variables such as distance, frequency of attendance at the health facility and employment status [8].

The Sickle Cell Unit (SCU), based in Kingston Jamaica, is the only SCD center in the English- speaking Caribbean, seeing more than 3,000 SCD patients annually. There is no published research on the knowledge, beliefs and practices of persons living with SCD regarding eye complications. The objective of this study was to determine the knowledge and beliefs regarding eye disease of adults attending the Sickle Cell Unit (SCU), Kingston, Jamaica and to assess whether these were associated with their practice. The impact of genotype, as well as demographic and socio-economic status on knowledge, belief and practice were assessed. This data can assist health care workers counsel patients with sickle cell disease in the Caribbean, Africa and other countries where the sickle cell gene is prevalent. Early referral, eye screening and treatment of sickle cell patients with sickle cell retinopathy can reduce the eye complications that lead to visual loss.

## Methods

A quantitative cross-sectional study was undertaken at the Sickle Cell Unit at the University of the West Indies, Jamaica during May 2016. Consecutive non-pregnant adults (>18 years of age) attendees, who were not acutely unwell, were invited to participate. A 26-item single interviewer administered questionnaire was used to obtain socio-demographic data (age, gender, parish of residence (In the Kingston Metropolitan Region or rural parishes), highest level of education completed (primary, secondary or tertiary), employment status, sickle cell genotype, if known, frequency of clinic attendance and patients' knowledge (seven questions), beliefs (six questions) and practices (five questions). Ten of these were yes/no questions, whereas eight required that they choose correct answers from four choices. Respondents who attended clinic one-two times per year were deemed adherent, while those who attended clinic less than once annually were deemed non-adherent. Those attending three or more times were deemed to be more symptomatic, requiring ad hoc visits. The questionnaire was based on an instrument previously used in Jamaica to assess knowledge, beliefs and practices regarding diabetic eye disease [7].

*Statistical analysis:* the sample size of 100 was determined with a 95% confidence level and 10, as the confidence interval with a population of 3000. Knowledge, belief and practice scores were computed, with one point given for each correct answer.

Written informed consent was obtained. Confidentiality and anonymity were ensured and the research protocol was approved by the Faculty of Medical Sciences, University of the West Indies/University Hospital of the West Indies Ethics Committee. Statistical analysis was by SPSS version 12.

## Results

One hundred subjects were recruited from the SCU clinics.

**Socio-demographic status:** fifty six (56%) were female. The mean age was 34.1 years (SD 11.3, range 18-63). Twenty percent of the subjects had Hb SC disease, whereas 72% and 2% reported Hb SS disease and Hb Sβ+ thalassemia respectively. Six percent were unaware of their genotype. The majority achieved secondary education (61%), whereas 14% had completed primary and 25% tertiary education. Most of the patients (62%) were unemployed. Three quarters lived within the Kingston Metropolitan Area. Seven attended clinic less than once a year and 42 attended more than twice yearly, while 51% attended 1-2 times yearly, as recommended for health maintenance visits. The median number of annual visits was two (range 0 to 50).

**Figure 1:**
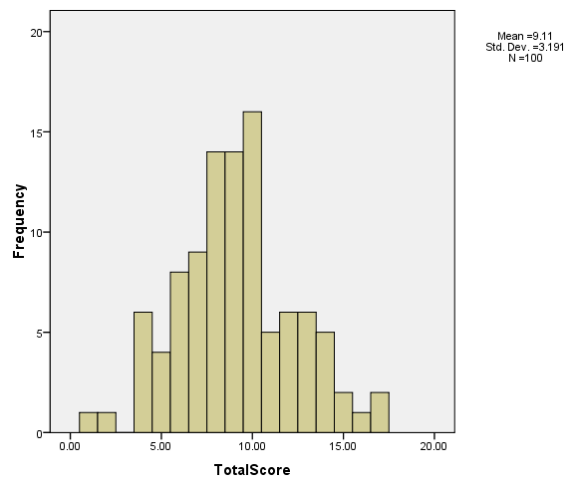
Distribution of the scores

**Knowledge, belief and practices scores:** the mean score was 10 (55%) (SD 3.5, range 1 to 18) and was normally distributed (Figure 1). The highest scoring category was in belief where 60% (mean score 3.6) were correct (Table 1). The lowest scoring category was in practice (44%, mean score 2.2). There was significant variation in how many subjects answered each question correctly (Table 1) and some varied by genotype. Most patients did not know that SCD can lead to loss of vision (75%), that early detection can prevent visual loss (69%) or that those with HbSC disease have a higher risk of severe eye disease (93%). Many thought being tested for glasses was an adequate eye examination (73%), and that SCD did not cause blindness. Whereas 81% would see an ophthalmologist if they saw floaters, only 25% had regular eye examinations; 75% only see an eye doctor if they are symptomatic and less than half had seen an ophthalmologist in the previous year. Knowledge scores were higher in those with milder genotypes than those with severe genotypes (4.0 versus 3.2, *t*=2.6, P=0.013). Patients with milder genotypes were more likely to know that HbSC is the genotype most closely associated with SCD related eye disease (18.2% versus 4.2%, χ^2^=4.8, P=0.05). Patients who lived within the Kingston Metropolitan Area, when compared with those who lived further from the clinic, had higher correct belief scores (3.8 (1.1) versus 3.1 (1.4), t=2.2, P=0.03), but there was no association between address and knowledge or practice scores. They were more likely to believe that some people with SCD will get associated eye disease (56.0% versus 32.0%, χ^2^=4.3, P=0.04). Those who lived within the Kingston Metropolitan Area appeared to be more likely to be adherent with clinic attendance, but this did not achieve statistical significance (96.0% versus 84.0%, χ^2^=4.1, P=0.06).

**Table 1:** Mean knowledge, belief and practices score and the % correct answers

Sections	% correct
**KNOWLEDGE (7 questions) Mean Correct (SD) 3.3 (1.4) Mean % correct 47%**	
Can having sickle cell disease affect the vision?	76%
Can you prevent loss of vision from sickle cell eye disease?	31%
Can patients with sickle cell disease have floaters?	39%
Should patients with sickle cell disease have regular eye examinations?	93%
Should patients with sickle cell disease see their eye doctor only when they have an eye problem?	62%
How does the sickle cell disease affect the vision?	25%
Which type of sickle cell disease (genotype) is more likely to be associated with severe eye disease?	7%
**BELIEF (6 questions) Mean Correct (SD) 3.6 (1.2) Mean % correct 60%**	
Do you think that a glasses test is an adequate eye test for a sickle cell patient?	27%
Do you think that patients with sickle cell disease should have a special eye test done?	85%
Do you believe that if you see an eye doctor regularly it will prevent you from losing vision?	76%
How often do you think that sickle cell disease affects the vision?	50%
What is the worse effect that sickle cell disease can have on the vision?	37%
How often do you think sickle cell patients should see their eye doctor?	90%
**PRACTICE (5 questions) Mean Correct (SD) 2.2 (3.5) Mean % correct 44%**	
Do you see your eye doctor for regular eye check-ups?	28%
Do you see your eye doctor only if you have a problem with your vision or your eyes?	25%
If you were having problems seeing, which medical person would you first visit?	39%
What would you do if you started to see black dots floating in your vision?	81%
When was the last time you saw an ophthalmologist (eye doctor)?	42%
**TOTAL (18 questions) Mean Correct (SD) 10 (3.5) Mean % correct 55%**	

Practice scores were higher in employed than in unemployed patients (2.6 versus 1.9, (t=2.1, P=0.04). Employed patients were more likely than the unemployed to see their eye doctor for regular eye examinations (42.1% versus 19.4%, χ^2^=6.0, P=0.02). Though not achieving statistical significance, there were trends towards an association of practice score with higher educational level (χ^2^=5.3, P=0.07) and correlation between practice score with increased age (r=0.19, P=0.06). None of the scores were significantly associated with gender. The Practice Score was correlated to both knowledge score (r^2^=0.363, P value<0.001) and belief score (r^2^=0.304, P value=0.002). However, 98% believed sickle cell patients should see an ophthalmologist at least once a year, however, only 42% had done so, and 21% reported never seeing an ophthalmologist. A model to predict Practice Score which included age, education, employment status, Knowledge and Belief scores explained 25% of the variability. Significant predictors were knowledge score (β=0.25, P value=0.02), education score (β=0.48, P value=0.04) and age (β=0.03, P value=0.02).

## Discussion

In Jamaica 1:150 births have a form of sickle cell disease, 1:300 births are homozygous HbSS disease and the frequency is not declining [9, 10]. With increased use of disease modifying therapies, there is increased life expectancies and reduced complications [11]. In Canada, eye screening for PSR has been recommended at 9 years of age for HbSC and 13 years of age for HbSS [12]. In Jamaica, 12 years age is the recommended age of screening [13]. Two decades ago, sight threatening PSR was most frequently seen in the 21-26 year old age group, however, it is now more prevalent in patients over 35 years old. Therefore, continued screening and early treatment for this disease is needed [4, 14]. It is important that SCD patients are knowledgeable about their disease and preventable complications and practice regular eye examinations as recommended.

In our study, the mean belief score was 60%, the mean knowledge and practice scores were below 50%. Compared to a study on Jamaican diabetic patients, which suggested that a knowledge score of >80% was adequate [7] this study highlights inadequate knowledge and inappropriate beliefs leading to practice which could decrease good eye care outcomes. Many participants were unable to correctly answer basic information regarding eye disease in SCD. This suggests that in these patients with SCD, despite attending the SCU, where the discussion of eye complications and referral for routine eye examination is routine, there is much room for improvement of their knowledge, beliefs and practices to avoid poor ocular outcomes. Demographic variables influenced practice. Although the possible relationship between age and educational level with practice did not reach statistical significance, being employed was associated with regular eye examinations. Limited access to public funded hospitals (no fee to the user) and the cost of travel to attend such appointments may deter unemployed individuals from receiving the necessary healthcare or preventative investigations [15]. The higher transportation costs associated with living further from the SCU may account for worse adherence in rural subjects, and this may have influenced their lower belief scores. In this study, there was no gender difference in knowledge scores, which contrasts with previous Jamaican studies in which women had better knowledge scores [7, 16] . The higher knowledge scores in subjects with milder genotype may reflect a counselling bias in the staff, as the prevalence and severity of eye disease is higher in Hb SC disease [4, 17]. This type of complication specific intervention is also needed for other complications of SCD. A lack of knowledge is also implicated in poor outcomes of priapism in men with SCD [15]. Care must be taken to design and test possible tools assessing KBP amongst patients. Many interventions to improve disease-specific knowledge in persons with SCD have been tested, but few have shown convincing benefit [18].

The prevalence of the sickle cell gene is high in Africa, in particular the Northern Tanzania, where it has been reported in 21.1% of births [19]. In a study from Morocco, sickle cell disease was the most common haemoglobinopathy with 23% having homozygous HbSS and additional 43.5% having the sickle gene [AS heterozygous AS (40.6%), S/ß-thalassemia composite heterozygosity (2.9%)] on a paediatric ward [20]. A cohort study from Kenya revealed that 69.3% of children that were admitted were undiagnosed sickle cell anemia patients [21]. This means that the true prevalence is unknown and there is a potential public health problem. A study from Togo revealed that Sickle cell disease is the cause of 16% of retinal detachments [22]. They concluded that sickle cell disease is a significant major risk factor(p=0.0003) for tractional retinal detachment, a type of retinal detachment that can be surgically challenging. [22]. Sickle Cell retinopathy can cause severe eye disease resulting in visual loss, and patients must be educated and encouraged to get early screening [23] . Because of the association between both knowledge and belief with practice, interventions changing both have potential to improve outcomes. Educational modules could be developed that could be integrated into usual care, targeting those who will benefit most. The use of a convenience sample in a single site may limit the generalizability of the study. However, many of the issues raised, such as unemployment, distance from care, lack of effective counselling tools and an increased risk of adverse outcomes are relevant to complications affecting adults living with SCD in many settings. Standard of care includes counselling about eye complications of SCD, referral of all individuals over the age of 12 years for biennial routine eye assessments and urgent attention for eye symptoms. It is important to improve the knowledge, beliefs and practices of all SCD patients in order to prevent the loss of vision from the ocular complications of sickle cell disease. Due to migration factors, countries, other than Africa, the Caribbean and the Mediterranean will need to ensure the standard of care of sickle cell patients, including regular eye screening [24].

## Conclusion

Sickle cell patients have a fair knowledge of the eye complications associated with SCD, however they don't usually get regular screening eye examination. Factors that impact on this poor correlate between knowledge and practice includes unemployment and distance from the hospital amongst other factors. Countries with a high prevalence of sickle cell disease, must ensure that adequate counselling and encouragement is done by health workers and policies put in place to ensure adequate regular eye screening is done for these patients.

### What is known about this topic

- No previous published study on sickle cell disease patient's knowledge, beliefs and practices on eye diseases related to SCD;
- There is a high prevalence of sickle cell disease in African countries;
- Sickle cell retinopathy can result in visual loss.

### What this study adds

- SCD patients need additional measures to increase counselling re eye diseases and what they need to do;
- Employed SCD patients and those who live closer to hospital are more likely to access eye care;
- Patients need to be educated about the guidelines for retinopathy screening for SCD.

## Competing interests

The authors declare no competing interests.
